# Assessing SARS-CoV-2 Rare Mutations and Transmission in New York City by NGS

**DOI:** 10.3390/microorganisms13081821

**Published:** 2025-08-04

**Authors:** Dakai Liu, Harlan Pietz, George D. Rodriguez, Yuexiu Wu, Yihan Cao, Vishnu Singh, Hui Li, Eric Konadu, Keither K. James, Calvin Lui, Bright Varghese, Mingyu Shao, Gary Chen, Andrew Schreiner, Jiankun Tong, Carl Urban, Nishant Prasad, Ameer Hassoun, Manish Sharma, William Harry Rodgers

**Affiliations:** 1 Department of Pathology and Clinical Laboratories, New York-Presbyterian Queens, 56-45 Main Street Flushing, New York, NY 11355, USA; dal9165@nyp.org (D.L.); harlan.pietz@yale.edu (H.P.);; 2 Weill Cornell Medical College, 1300 York Avenue, New York, NY 10065, USA; 3 Department of Quality and Patient Safety, New York-Presbyterian Queens, 56-45 Main Street Flushing, New York, NY 11355, USA; gdr9005@nyp.org; 4 Division of Infectious Disease, New York-Presbyterian Queens, 56-45 Main Street Flushing, New York, NY 11355, USA; wuy@thewrightcenter.org; 5 Massachusetts General Hospital, Harvard Medical School, 55 Fruit Street, Boston, MA 02114, USA; 6 Department of Emergency Medicine, New York-Presbyterian Queens, 56-45 Main Street Flushing, New York, NY 11355, USA; 7 Department of Pathology and Laboratory Medicine, Weill Cornell Medical College, 1300 York Avenue, New York, NY 10065, USA

**Keywords:** SARS-CoV-2, COVID-19, next-generation sequencing, mutation, viral variants

## Abstract

SARS-CoV-2 undergoes frequent mutations that drive viral evolution and genomic diversity, influencing transmissibility, immune escape, and disease severity. In this study, we performed whole-genome sequencing on SARS-CoV-2 isolates from patients in New York City and identified several globally rare mutations across multiple viral lineages. The isolates analyzed for rare mutations belonged to three lineages: B.1.1.7 (Alpha), B.1.526 (Iota), and B.1.623. We identified 16 rare mutations (global incidence <1000) in non-structural protein genes, including *nsp2*, *nsp3*, *nsp4*, *nsp6*, *nsp8*, *nsp13*, *nsp14*, *ORF7a*, and *ORF8*. Three of these mutations—located in *nsp2, nsp13,* and *ORF8*—have been reported in fewer than 100 individuals worldwide. We also detected five rare mutations in structural proteins (*S*, *M*, and *N*), including two—one in *M* and one in *N*—previously reported in fewer than 100 cases globally. We present clinical profiles of three patients, each infected with genetically distinct viral isolates from the three lineages studied. Furthermore, we illustrate a local transmission chain inferred from unique mutation patterns identified in the Omicron genome. These findings underscore the importance of whole-genome sequencing for detecting rare mutations, tracking community spread, and identifying emerging variants with clinical and public health significance.

## 1. Introduction

Since late 2019, COVID-19 has resulted in over 770 million confirmed cases and more than 7 million deaths worldwide [1]. The causative agent, severe acute respiratory syndrome coronavirus 2 (SARS-CoV-2), belongs to the *Coronaviridae* family [2]. Its positive-sense, single-stranded RNA genome is approximately 29.9 kilobases (kb) in length and encodes sixteen non-structural proteins (NSP1–NSP16), along with four structural proteins: the nucleocapsid (N), envelope (E), membrane (M), and spike (S) glycoprotein [3].

The gold standard for SARS-CoV-2 detection in clinical specimens is multiplex reverse transcription polymerase chain reaction (mRT-PCR), which amplifies two or more viral nucleic acid targets using pathogen-specific primer/probe sets [4]. Combined with patient-specific risk factors and clinical presentation, mRT-PCR results guide treatment decisions and isolation protocols in both inpatient and outpatient settings [2].

Like other RNA viruses, SARS-CoV-2 exhibits a high mutation rate during replication [4]. Since the first viral genome sequence published in early 2020, more than 150,000 mutations have been identified globally, leading to the classification of over 1600 lineages or variants [5]. Some of these, designated as variants of concern (VOCs), have shown increased transmissibility, immune evasion, or pathogenicity. These include Alpha (B.1.1.7 and sub-lineages) [6,7], Delta (B.1.617.2 and sub-lineages) [8], and Omicron (BA.1/BA.2 and related sub-lineages) [9].

In our earlier work, we applied genomic sequencing to monitor SARS-CoV-2 genetic diversity in New York City, enabling early detection of VOCs within the local population [4]. By comparing viral genomes across regions and time points, we reconstructed transmission chains and identified superspreading events, providing insights into how the virus spreads through communities [10]. Additionally, our genomic data helped refine molecular diagnostic assays, ensuring their continued accuracy in the face of viral evolution [4]. These previous studies underscored the critical role of viral genome sequencing in supporting evidence-based public health decision-making and outbreak response.

In this report, we investigate the prevalence and phylogenetic relationships of SARS-CoV-2 clinical isolates collected from patients in New York City. Our analysis focuses on identifying rare nonsynonymous mutations and mapping them within viral protein structures to explore their potential functional implications. We also characterize local community transmission by tracing unique mutation patterns within the viral genome. By integrating genomic, structural, and clinical data, we provide a comprehensive, longitudinal perspective on the COVID-19 pandemic. This multifaceted approach offers valuable insight into population-level transmission trends, individual patient outcomes, and the molecular evolution of SARS-CoV-2 in one of the most severely impacted centers in the United States.

## 2. Materials and Methods

### 2.1. Sample Collection

Between 6 March 2020 and 15 April 2023, 283,083 nasopharyngeal swab specimens were collected from patients at NewYork-Presbyterian Queens for SARS-CoV-2 RNA testing by diagnostic multiplex real-time PCR. A total of 18,716 serum specimens were collected between 10 April 2020 and 8 April 2023, for measuring anti-SARS-CoV-2 total antibodies.

### 2.2. SARS-CoV-2 RNA Detection

Nasopharyngeal swab specimens were assayed by multiplex reverse transcription PCR (mRT-PCR) for the presence of SARS-CoV-2 RNA using the Cepheid^®^ Xpert Xpress SARS-CoV-2 assay on the GeneXpert Infinity system (Cepheid, Inc., Sunnyvale, CA, USA). A positive result from this assay yields two amplicons with specific sets of primers and probes. The first amplicon targets a region in the nucleocapsid (N) gene unique to SARS-CoV-2. The second amplicon targets a conserved region of the envelope (E) gene present in all Sarbecoviruses. Each assay included a sample processing control and a probe check control. The analytical sensitivity of the assay was determined by serial dilutions of known concentrations of ZeptoMetrix NATSARS(CoV2)-ERC virus stock. The limit of detection was determined to be 30 virions per assay.

### 2.3. Anti-SARS-CoV-2 Total Antibody Detection

Total anti-SARS-CoV-2 antibodies were measured via a sandwich electrochemiluminescence immunoassay (Roche Elecsys anti-SARS-CoV-2, Roche Diagnostics, Indianapolis, IN, USA) on a Roche Cobas e 411 immunoassay analyzer. Serum specimens were first incubated with biotinylated SARS-CoV-2 recombinant antigen with or without ruthenium labeling [11] to form a sandwich complex. This biotinylated complex is rendered into a solid phase through the addition of streptavidin-coated microparticles, and the reaction mixture is subsequently immobilized to an electrode. Following a wash step, chemiluminescent emission of immobilized antibody–antigen complexes is quantified with a photomultiplier [11]. Comparison of the electrochemiluminescence signal obtained from the reaction product of the sample with the signal of the calibration cutoff value was used to determine results automatically through the cobas e 411 software version 03-02 [11].

### 2.4. Viral Genomic Next-Generation Sequencing and Bioinformatics

SARS-CoV-2 mRT-PCR-positive nasopharyngeal swab specimens with a real-time PCR cycle threshold (Ct) value of <33 cycles were subjected to next-generation sequencing (NGS) using an Illumina COVID-Seq test kit. RNA was extracted from nasopharyngeal swab specimens in viral transport media, and cDNA synthesis was performed through reverse transcription with random hexamer primers. cDNA fragments were amplified through two separate PCR reactions, and products were pooled together before being subjected to bead-based adaptor tagmentation. Adaptor-tagged fragments were subjected to another round of PCR amplification before pooling and cleaning of the index-tagged libraries with purification beads. Pooled libraries were clustered onto a flow cell and sequenced on the NovaSeq 6000 system (Illumina Instruction for Use, Illumina Inc., Hayward, CA, USA). Sequence analysis was performed with VarSeq version 2.2.2 (Golden Helix, https://www.goldenhelix.com, accessed on 13 June 2021), and the consensus sequence for each isolate was input to Nextclade version 1.10.1 for quality control, mutation calling, and Nextstrain clade assignment. Isolates with sequences <29,000 nucleotides in length or with a Nextclade-assessed “qc.overallStatus” below “good” were considered low quality and removed [12]. Nucleotide changes in the 3′ end of the genome, including those in ORF10 and the terminal 50 amino acids of the N gene, were assessed as artifacts of the sequencing process and were not analyzed as true mutations.

To assess mutation frequency, 1,444,000 available SARS-CoV-2 genomes and associated metadata, uploaded by 13 June 2021 to the Global Initiative on Sharing All Influenza Data (GISAID), were downloaded on 6 July 2021 and analyzed [13,14,15]. Sequences were aligned by MAFFT [16]. Individual mutations in this study are specified by the notation {SARS-CoV-2 gene} [amino acid position]/(residue change):GISAID frequency. In addition, we conducted an analysis of rare mutations previously identified using the updated GISAID database up to 22 July 2025.

## 3. Results

### 3.1. Prevalence of Positive SARS-CoV-2 mRT-PCR and Anti-SARS-CoV-2 Antibody Test Results

NewYork-Presbyterian Queens Hospital was at the epicenter of the COVID-19 pandemic and received 283,083 specimens for SARS-CoV-2 RNA testing using diagnostic multiplex real-time PCR between 6 March 2020 and 6 April 2023. Of these, 24,135 specimens (8.53%) tested positive. The specimens were collected from 171,359 unique individuals, some of whom were tested more than once. Among these individuals, 19,565 were confirmed to have SARS-CoV-2 infection, yielding an average incidence rate of 14.42%. In addition, the hospital tested 18,716 specimens for total anti-SARS-CoV-2 antibodies between 20 April 2020 and 8 April 2023, of which 6197 (33.1%) were positive.

### 3.2. Prevalence, Phylogeny, and Domain Localization of SARS-CoV-2 Mutations in the B.1.1.7 (Alpha) Lineage

**Figure 1:**
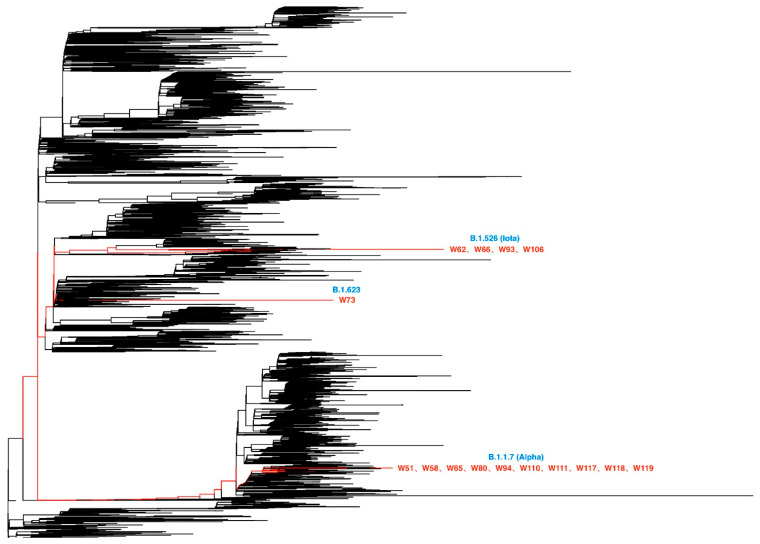
Phylogenetic tree of SARS-CoV-2 virus isolates analyzed for mutations. This phylogenetic tree shows the relationships among SARS-CoV-2 variants isolated between December 2020 and March 2021. Lineages are labeled in blue, and the corresponding isolates are listed in red beneath each lineage. Ten isolates (W51, W58, W65, W80, W94, W110, W111, W117, W118, and W119) belong to the B.1.1.7 (Alpha) lineage. Four isolates (W62, W66, W93, and W106) are part of the B.1.526 (Iota) lineage. One isolate, W73, belongs to the B.1.623 lineage. Black branches represent contextual SARS-CoV-2 genomes from the GISAID database (https://www.gisaid.org, accessed on 13 June 2021).

**Figure 2:**
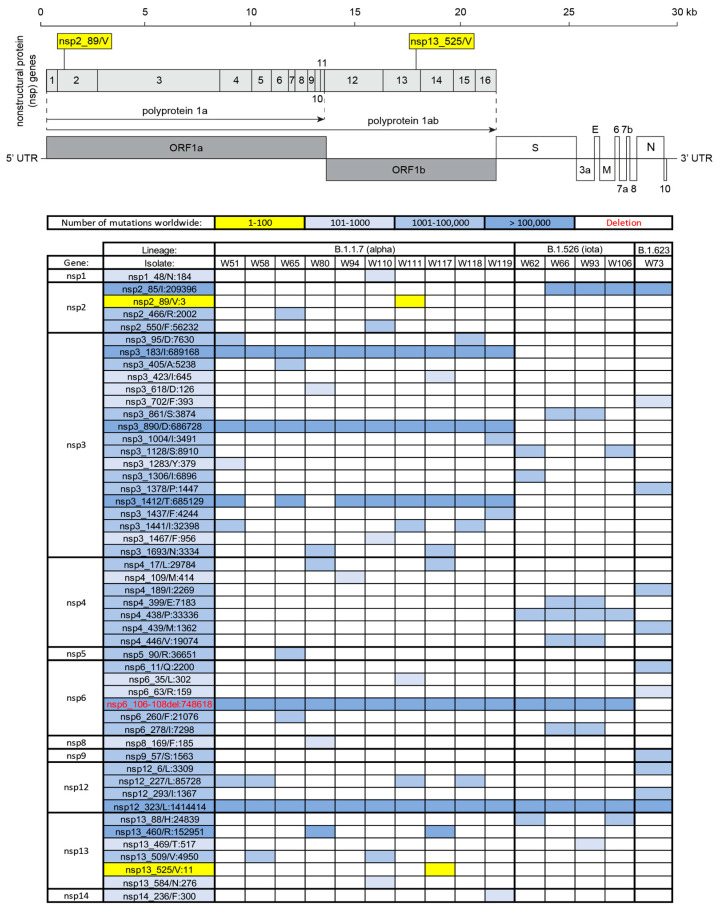
Mutation analysis of SARS-CoV-2 non-structural proteins in polyprotein 1a and 1b. The diagram at the top illustrates the positions of non-structural proteins (nsp1–nsp16) encoded by the ORF1a and ORF1b regions of the SARS-CoV-2 genome. Mutation frequencies across the global SARS-CoV-2 population are shown using a blue color scale, with deletions highlighted in red. The table below summarizes mutation frequencies across different genes observed in each isolate, with rare mutations highlighted in yellow.

**Figure 3:**
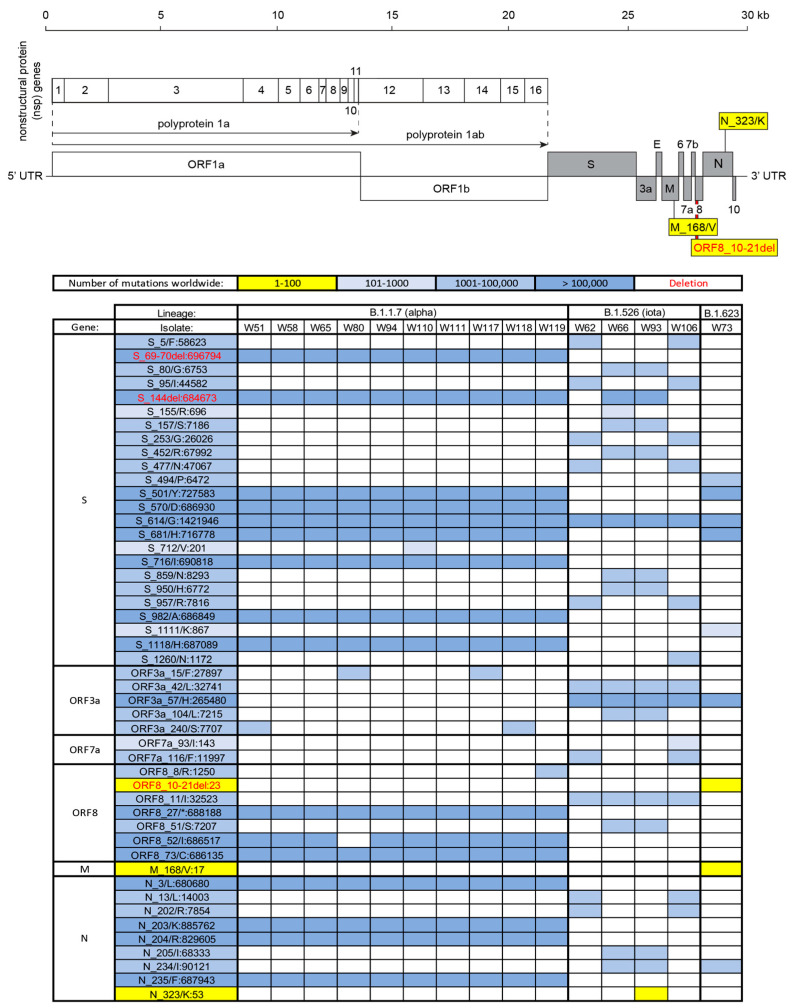
Mutation analysis of SARS-CoV-2 gene structure proteins and accessory open reading frames (ORFs). The diagram at the top shows the genomic organization of SARS-CoV-2 structural proteins (S, E, M, and N) and accessory proteins encoded by ORFs 3a, 6, 7a, 7b, 8, and 10. Mutation frequencies observed globally are indicated using a blue color gradient, with deletions shown in red. The table below summarizes the mutation frequencies across different genes for each isolate. Rare mutations are highlighted in yellow.

To investigate the continuous genetic divergence of SARS-CoV-2 variants, viral genomic RNA from 9516 SARS-CoV-2 mRT-PCR-positive specimens were isolated and sequenced. After filtering by sequence quality, 7586 (79.7%) of the isolates were analyzed for mutations. Of these isolates, fifteen with deep sequencing data were identified as having one or more globally (<1000 worldwide) and/or locally (<100 in New York State) rare nonsynonymous mutations. Ten of these fifteen isolates (specimens from W51, W58, W65, W80, W94, W110, W111, W117, W118, and W119) belonged to the B.1.1.7 (Alpha) lineage (Figure 1), which shares a set of three defining deletion mutations in two separate SARS-CoV-2 genes: (1) a three amino acid deletion in the non-structural protein 6 (nsp6) gene (nsp6_106-108del) (Figure 2), (2) a two amino acid deletion in the spike (S) glycoprotein gene (S_69-70del) (Figure 3), and (3) a second, single amino acid deletion in the spike (S) glycoprotein gene (S_144del) (Figure 3). Additional B.1.1.7 (Alpha) lineage-defining, nonsynonymous mutations were also present throughout all 10 isolates, including S_501/Y, S_570/D, S_614/G, S_681/H, S_716/I, S_982/A, S_1118/H, ORF8_27/*, ORF8_73/C, N_3/L, and N_235/F (Figure 3) [17,18,19]. These mutations were identified from the isolates collected during the period from December 2020 to March 2021. Up to 22 July 2025, 5 rare mutations (nsp2_89/V, nsp13_525/V, ORF8_10-21del, M-168/V, and N_323/K) identified before are still considered rare with approximately 17 million isolates analyzed, which are available in GISAID.

### 3.3. Genomic Diversity of the nsp3 Gene in the B.1.1.7 (Alpha) Lineage

Among isolates from the SARS-CoV-2 B.1.1.7 (Alpha) lineage, most rare mutations were in genes of non-structural proteins. Of these, the non-structural protein nsp3—which at ~200 kDa is the largest protein encoded by the SARS-CoV-2 genome—bore the highest number of mutations [14] across the ten B.1.1.7 (Alpha) lineage isolates, excluding the B.1.1.7 (Alpha) lineage-defining nsp3 mutations nsp3_183/I, nsp3_890/D, and nsp3_1412/T. Included among these were globally rare nsp3 mutations identified in isolates W51 (nsp3_1283/Y:379), W80 (nsp3_618/D:126), W110 (nsp3_1467/F:956), and W117 (nsp3_423/I:645).

Two isolates—W58 and W80—lacked the B.1.1.7 (Alpha) lineage-defining nsp3_1412/T mutation at the C-terminal (ER-proximal) end of the TM1 domain of Nsp3 (Figure 2). The nsp3_1467/F:956 mutation is in the Nsp3 ectodomain (3Ecto). This domain, situated between the two transmembrane (TM) domains (TM1 and TM2) of Nsp3, which anchor the protein to ER-derived double-membrane vesicles, is predicted to be the only Nsp3 domain localized to the ER luminal compartment. The interactions of 3Ecto with a loop of Nsp4 are essential for the assembly of ER-origin double-membrane vesicles required for SARS-CoV-2 replication [20].

### 3.4. Other Rare and Noteworthy Mutations Among Isolates from the B.1.1.7 (Alpha) Lineage

The SARS-CoV-2 gene with the second-most mutations [4] identified among isolates in the B.1.1.7 (Alpha) lineage was nsp13. The gene product of nsp13 (Nsp13) is a helicase essential for SARS-CoV-2 replication that unwinds either DNA or RNA substrates [21]. Nsp13 interfaces with the replication–transcription complex (RTC), which is composed of Nsp12 (the RNA-dependent RNA polymerase, or RdRP), Nsp7, and two copies of Nsp8, to synthesize progeny RNA from the SARS-CoV-2 RNA genome [22]. RNA synthesis by the RTC and Nsp13 is assisted by several nucleic acid-processing enzymes, including Nsp14 (an exonuclease/methyltransferase), Nsp15 (an endonuclease), and Nsp16 (another methyltransferase) [23]. One isolate—W117—featured a globally rare nsp13 mutation (nsp13_525/V:11). This mutation is in the Nsp13 2A domain, which forms part of the RNA-binding channel of Nsp13 along with the 1A and 1B domains [24]. Another isolate—W110—also featured a globally-rare mutation (nsp13_584/N:276) in the Nsp13 2A domain.

The SARS-CoV-2 gene with the third-most mutations [6] identified among isolates in the B.1.1.7 (Alpha) lineage was nsp2. The Nsp2 protein may interfere with cellular immune responses by indirectly suppressing the immunostimulatory cytokine interferon beta (IFN-β) [25]. One isolate—W111—featured a globally rare mutation (nsp2_89/V:3) observed in the nsp2 N-terminal domain between two zinc finger motifs involved in nucleic acid binding [26]. Isolate W111 also featured a globally rare mutation near the N-terminus of the nsp6 gene (nsp6_35/L:302). Like Nsp3 and Nsp4, Nsp6 is essential for assembling the double-membrane vesicular architecture sourced from ER membranes, with Nsp6 as a molecular “zipper” [20].

Two mutations were observed among the nsp4 gene among isolates from the B.1.1.7 (Alpha) lineage, including one globally rare mutation (nsp4_109/M:414) in isolate W94, which is predicted to be localized to the ER lumen [27]. One globally rare mutation was observed in the N-terminal portion of nsp1 (isolate W110, nsp1_48/N:184). During SARS-CoV-2 infection, the C-terminal portion of the Nsp1 protein inhibits host cell translation by physically plugging the mRNA entry channel in the 40S ribosomal subunit. The N-terminal portion of Nsp1 competes with early initiation factor 3j (eIF3j) binding to the uS3 protein in the 40S ribosomal subunit [28]. One globally rare mutation was also observed in both nsp14 (isolate W119, nsp14_236/F:300, situated in the exonuclease domain) and nsp8 (isolate W80, nsp8_169/F:185, located near the C terminus). Notably, isolate W80—which featured the only nsp8 mutation among B.1.1.7 (Alpha) lineage isolates in this dataset—lacked the Alpha lineage’s ORF8_52/I mutation characteristic (Figure 3). This isolate, which was collected from a 72-year-old man who passed away from sepsis while hospitalized for COVID-19 pneumonia, also featured two distinguishing nsp3 mutations, as discussed previously: the globally rare nsp3_618/D:126 mutation and the absence of the B.1.1.7 (Alpha) lineage-defining mutation nsp3_1412/T. Only one isolate among the B.1.1.7 (Alpha) lineage featured a globally rare mutation in a gene for a structural protein. Isolate W110 bore a mutation in the spike glycoprotein S2 subunit (S_712/V, 201 sequences worldwide).

### 3.5. Prevalence, Phylogeny, and Domain Localization of SARS-CoV-2 Mutations in the B.1.526 (Iota) Lineage

Unlike the SARS-CoV-2 B.1.1.7 (Alpha) lineage, mutations in structural genes—particularly in the spike (S) glycoprotein—were more common in the four isolates from the B.1.526 (Iota) lineage (Figure 3). Excluding the S_614/G mutation, which was present in all 15 SARS-CoV-2 isolates in this study, twelve distinct mutations in the S gene were identified among the four isolates of the B.1.526 (Iota) lineage. Indeed, the four isolates in the B.1.526 (Iota) lineage may be divided into two pairs. The first pair of isolates (W62 and W106) featured three common B.1.526 (Iota) lineage-defining mutations in the S gene, S_5/F:58623, S_95/I:44582, and S_253/G:26026, which were absent in the second pair of isolates (W66 and W93). Isolates W62 and W106 also feature two additional common mutations—S_477/N:47067 and S_957/R:7816—that were also not present in isolates W66 and W93. The S_1260/N:1172 mutation was only present in isolate W106. The second pair of isolates (W66 and W93) featured five common S gene mutations—S_80/G:6753, S_157/S:7186, S_452/R:67992, S_859/N:8293, and S_950/H:6772—that were not found in isolates W62 and W106. Isolate W66 also featured a globally rare mutation in the S gene (S_155/R:696) located in the S1 subunit.

Isolate W93 featured a globally rare mutation in the C-terminal domain of the N gene (N_323/K:53) (Figure 3). A multi-domain RNA binding protein, the N protein wraps the SARS-CoV-2 genomic RNA into a helical structure during virion assembly and interferes with RNA-directed host antiviral responses [29]. Isolate W93, which was obtained from a 63-year-old man who passed away from sepsis while hospitalized for COVID-19 pneumonia, also featured a globally rare mutation in the 2A domain that forms part of the RNA-binding channel of the nsp13 gene (nsp13_469/T:517). One globally rare mutation (ORF7a_93/I:143) in the non-structural protein ORF7a was identified in isolate W106. The ORF7a protein of SARS-CoV-2 is an antagonist of multiple host antiviral proteins and reduces antigen presentation via the MHC-I pathway [30,31].

### 3.6. Prevalence, Phylogeny, and Domain Localization of SARS-CoV-2 Mutations in a Single Isolate from the B.1.623 Lineage

Only one isolate from our set—W73—did not belong to either the B.1.1.7 (Alpha) or B.1.526 (Iota) lineages. Isolate W73 was obtained from a 27-year-old male who experienced comorbid bacterial pharyngitis while infected with SARS-CoV-2 but did not require hospitalization. A member of the B.1.623 lineage, isolate W73 featured a rare deletion of 12 amino acids in ORF8 (ORF8_10-21del:899) (Figure 3). Isolate W73 also featured globally rare mutations in non-structural proteins nsp3 (nsp3_702/F:393) and nsp6 (nsp6_63/R:159) (Figure 2). Isolate W73 featured globally rare mutations in the S2 domain of the S gene (S_1111/K:867) and in the C-terminal domain of the M gene (M_168/V:17) (Figure 3). The most abundant protein in the SARS-CoV-2 viral envelope, the homodimeric M protein, plays a critical role as a scaffold for assembling other structural proteins into the mature SARS-CoV-2 virion [32].

### 3.7. Mutation Analysis and Community Transmission of Omicron

In addition to characterizing rare mutations across various SARS-CoV-2 lineages, our study examined the relationship between viral genetic changes and epidemiological patterns of community transmission during the early Omicron wave in New York City. Specifically, we investigated clusters of SARS-CoV-2 infections within ZIP code regions, with a focus on identifying localized transmission events using next-generation sequencing (NGS) and accompanying demographic data.

Between 25 November and 11 December 2021, a subset of Omicron-positive clinical samples was selected for whole-genome sequencing and geographic linkage analysis. Using patient residential and workplace addresses—matched to ZIP code-level epidemiological data—we identified instances of apparent community transmission involving individuals living or working in close proximity. Several cases were traced to shared physical environments, such as apartment buildings or workplace settings, highlighting the potential for localized outbreak clusters.

As shown in Table 1, three distinct clusters of transmission were identified:

- Household A: Two individuals (nyo-mi029 and nyo-mi275), residing in the same apartment complex in ZIP code 10024, tested positive within six days of each other.
- Workplace A: Two cases (nyo-mi105 and nyo-mi231) from ZIP code 10001 were epidemiologically linked to the same business location, suggesting occupational exposure as the likely transmission route.
- Household B: Two additional cases (nyo-mi333 and nyo-mi342) from ZIP code 10024 also shared a residential address and tested positive on the same day, further supporting the occurrence of intra-household transmission.

**Table 1:** Genomic and epidemiological characteristics of Omicron community transmission cases.

	ID	Collection Date	Clade	Zip Code	Epidemiologic Features
1	nyomi029	12 March 2021	A	10024	Household A
2	nyomi275	12 September 2021	A	10024	Household A
3	nyomi105	12 August 2021	B	10001	Workplace A
4	nyomi231	12 September 2021	B	10001	Workplace A
5	nyomi333	12 October 2021	D	10024	Household B
6	nyomi342	12 October 2021	D	10024	Household B

**Figure 4:**
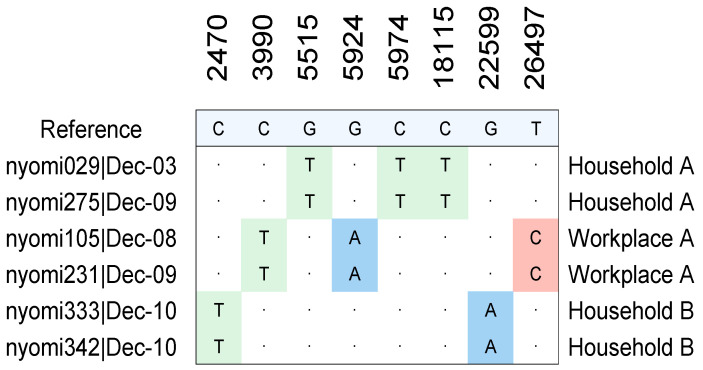
Mutational profiles of the SARS-CoV-2 Omicron variant. Each column corresponds to a specific genomic position, while color-coded shading indicates nucleotide substitutions: blue for adenine (A), red for cytosine (C), green for thymine (T), and light blue for guanine (G). Dots indicate no nucleotide substation.

To further illustrate the transmission dynamics, Figure 4 presents the mutational profiles of the Omicron variant sequences associated with these epidemiologically linked cases. The figure displays nucleotide substitution patterns across the viral genomes. Genomes isolated from patients living in the same household or working at the same location showed highly similar or identical mutational signatures, consistent with direct transmission or exposure to a shared source. These findings underscore the value of integrating genomic and epidemiological data to uncover hidden chains of transmission that may not be evident from case numbers alone. Such high-resolution genomic surveillance can inform targeted interventions in densely populated urban environments and enhance public health responses during periods of variant emergence.

## 4. Discussion

In this study, we performed whole-genome sequencing of SARS-CoV-2 clinical isolates obtained from patients who tested positive using a commercial RT-PCR assay. Our analysis revealed numerous mutations, some of which occur at low global frequencies. Notably, we identified a globally rare N gene mutation (N_323/K:53) in a fatal case involving a patient infected with a SARS-CoV-2 B.1.526 (Iota) lineage isolate. Prior studies suggest that mutations in the N gene may enhance viral replication and pathogenicity [3,9,27,29,33], raising concerns about their potential impact on disease severity and transmissibility. The clinical significance of the N_323/K:53 mutation warrants further investigation, particularly in the context of severe disease outcomes.

Additional rare mutations were identified in two other cases. Patient W80, infected with the B.1.1.7 (Alpha) lineage, carried the nsp3_618/D:126 and nsp8_169/F:185 mutations. Patient W73, infected with a B.1.623 lineage virus, exhibited multiple unique mutations, including nsp3_702/F:393, nsp6_63/R:159, S_1111/K:867, and M_168/V:17. These findings reflect the ongoing genetic diversification of SARS-CoV-2 and underscore the importance of continued genomic surveillance to detect and assess emerging mutations.

The multifunctional non-structural protein 3 (Nsp3) consists of 16 domains and is associated with the formation of double-membrane vesicles originating from the endoplasmic reticulum [34]. The nsp3_1283/Y:379 mutation is located between macrodomain 1 (Mac1 or X) and macrodomain 2 (Mac2 or SUD-N). Mac1 has been implicated in antagonizing host innate immune responses [35], while Mac2 plays a role in RNA binding [34]. The nsp3_423/I:645 mutation lies within Mac2, and the nsp3_618/D:126 mutation is situated in macrodomain 3 (Mac3 or SUD-M), which also participates in RNA binding [34]. These domain-specific mutations may alter viral replication or interactions with the host and should be further examined functionally.

This study is not without limitations. Since we did not perform functional assays to investigate the biological effects of the rare mutations identified, their pathogenicity and clinical relevance cannot be conclusively determined. Further functional studies are necessary to elucidate their potential roles in disease development and progression.

Given the heterogeneous clinical presentations of COVID-19 and the varying levels of immunity across populations, genomic surveillance using NGS remains crucial from both epidemiological and clinical perspectives. Understanding the genetic characteristics of emerging variants provides valuable insights into viral behavior, informs public health strategies, and supports the effective allocation of healthcare resources. Identifying mutations associated with increased transmissibility or severity can also help predict disease progression, guide treatment decisions, and improve patient management.

Moreover, genomic surveillance plays a vital role in reconstructing transmission chains within communities by identifying and tracking specific mutations in the viral genome [36,37]. As SARS-CoV-2 replicates, it naturally accumulates mutations. While many of these are neutral, some become distinctive markers of particular viral lineages or sub-lineages. By sequencing viral samples from infected individuals and comparing their genetic profiles, we can assess how closely related different infections are. This approach enables the mapping of transmission pathways. Phylogenetic analysis can reveal the direction and timing of transmission events, helping to distinguish between local spread and new introductions from outside the community. When combined with epidemiological data, genomic information can confirm or refute suspected links between cases, enhancing traditional contact tracing efforts.

Furthermore, genomic surveillance can identify clusters of infection. When multiple individuals share a unique set of mutations, it suggests a common source or a recent chain of transmission, allowing public health officials to pinpoint and respond to outbreak clusters more effectively.

Whole-genome amplification (WGA) and next-generation sequencing (NGS) have proven invaluable in the identification and characterization of SARS-CoV-2 variants. As demonstrated in this study and others, genomic analysis plays a pivotal role in monitoring viral evolution, mapping transmission dynamics, and shaping public health policy. Ongoing surveillance is also critical for evaluating the reliability of diagnostic assays, particularly in assessing the performance of primer/probe sets used in mRT-PCR testing. Continued research into SARS-CoV-2 genomic diversity will be essential for informing vaccine development, maintaining diagnostic accuracy, and enhancing preparedness for future viral mutations.

## Data Availability

The original contributions presented in this study are included in the article. Further inquiries can be directed to the corresponding author.

## Abbreviations

SARS-CoV-2	Severe acute respiratory syndrome coronavirus 2
COVID-19	Coronavirus disease of 2019
NSP	Non-structural protein
N	Nucleocapsid protein
E	Envelope protein
M	Membrane protein
S	Spike glycoprotein
mRT-PCR	Multiplex reverse transcription polymerase chain reaction
WGA	Whole-genome amplification
NGS	Next-generation sequencing
Ct	Cycle threshold
GISAID	Global Initiative on Sharing All Influenza Data
TM	Transmembrane
ORF	Open reading frame
